# Epidemiological Analysis of HTLV-1 and HTLV-2 Infection among Different Population in Central China

**DOI:** 10.1371/journal.pone.0066795

**Published:** 2013-06-24

**Authors:** Yunyun Ma, Shangen Zheng, Na Wang, Yu Duan, Xinyu Sun, Jing Jin, Wenqiao Zang, Min Li, Yuanyuan Wang, Guoqiang Zhao

**Affiliations:** 1 Department of Immunology & Microbiology, Basic Medical College, Zhengzhou University, Zhengzhou, Henan, China; 2 Department of Immunology & Microbiology, Henan Medical College for Staff and Workers, Zhengzhou, Henan, China; 3 Department of Blood Transfusion, Wuhan General Hospital of Guangzhou Military, Wuhan, Hubei, China; 4 Clinical Laboratory, The First Affiliated Hospital of Zhengzhou University, Zhengzhou, Henan, China; 5 Laboratory Department, Zhengzhou Central Hospital, Zhengzhou, Henan, China; Institut Pasteur, France

## Abstract

**Background:**

HTLV-1 and HTLV-2 are retroviruses linked etiologically to various human diseases, and both of them can be transmitted by vertical route, sexual intercourse, blood transfusion and intravenous drug use. Recently, some HTLV-infected cases have been reported and this virus is mainly present in the Southeast coastal areas in China, but has not been studied for the people in Central China.

**Objectives:**

To know the epidemiologic patterns among different population samples in Central China and further identify risk factor for HTLV-1 and HTLV-2 infection.

**Methods:**

From January 2008 to December 2011, 5480 blood samples were screened for HTLV-1/2 antibodies by using enzyme immunoassay, followed by Western Blot.

**Results:**

The prevalence of HTLV-1 and HTLV-2 was found with infection rates 0.13% and 0.05% among all population samples for HTLV-1 and HTLV-2, respectively. The highest percentages of infection, 0.39% and 0.20%, were found in the high risk group, while only 0.06% and 0.03% in the blood donor group. There was only one case of HTLV-1 infection (0.11%) among patients with malignant hematological diseases. Of seven HTLV-1 positive cases, six were co-infected with HBV, two with HCV and one with HIV. Among three HTLV-2 positive individuals all were co-infected with HBV, one with HCV.

**Conclusions:**

HTLV-1 and HTLV-2 have been detected in the Central China at low prevalence, with the higher infection rate among high risk group. It was also found that co-infection of HTLV-1/2 with HIV and HBV occurred, presumably due to their similar transmission routes. HTLV-1/2 antibody screen among certain population would be important to prevent the spread of the viruses.

## Introduction

Human T-cell lymphotropic virus (HTLV) was the first retrovirus discovered in humans [1]. HTLV-1 and HTLV-2 share approximately 70% homology at genetic level [2]. The two major pathologies associated with HTLV-1 infection and present in all endemic areas are: adult T-cell leukemia (ATL) and HTLV-1 associated myelopathy/tropical spastic paraparesis (HAM/TSP) [1], [3]–[5]. HTLV-1 has also been linked to cases of uveitis, infective dermatitis, polymyositis, synovitis, thyroiditis, and bronchioalveolar pneumonitis [6]. Although HTLV-2 has also been occasionally linked to neurological syndromes [7], [8], the majority of carriers remain asymptomatic lifelong. Recently, two novel HTLVs, HTLV-3 and HTLV-4, have been characterized [9]–[11]. However, only a few cases have been reported and no specific illnesses have yet been associated with these viruses [12].

HTLV-1/2 are present in different high-risk populations and spread globally. HTLV-1 is endemic in regions of the Caribbean, Central and Western Africa, Southwestern Japan, South and Central America, Melanesia and Australia. HTLV-2 has a more restricted distribution than HTLV-1, more prevalent among some native Americans and some Central African tribes, but is relatively common among intravenous drug users (IDUs) and their sex partners in Europe, North America, and other regions of the world [6], [8], [13]–[18]. HTLV-1/2 infections can be transmitted by vertical route (mother-to-child and breast milk), sexual intercourse and parental (blood transfusion and intravenous drug use) [19]. Over 20 million persons are infected with HTLV-1/2 in the world [20], but unfortunately, no vaccine is available against HTLV infection at the current time. The implementation of prevention measures after the antibody status determination of the HTLV prevalence level in a population, as the blood donors, is very important. Mandatory screening of blood supplies for HTLV-1/2 was implemented in the mid-1980s in countries such as Japan, Canada, and the United States and was being gradually established in many other countries.

China is considered to be a non-endemic region for HTLV as few positive individuals found in China. Since 1985, several investigations of the prevalence of HTLV have been carried out in China [21], [22]. All HTLV-1 seropositive individuals originate from the Fujian province in the Southeast with the prevalence rate 0.06% to 1.27% [20], [22], [23]. The Eastern coastal city of Fujian province, Xiamen, which is located in the highly endemic areas for HTLV,began to test HTLV-1/2 antibodies in blood donors in 2004 and became the first city in China which adopts this strategy. For the global Chinese population size is so huge and the tests for HTLV-1/2 mostly are performed in few Chinese regions, there may be positive individuals not found in other regions.

Henan and Hubei province, located in central areas of China, with large and great migrant population, are high endemic areas for blood transmitted diseases. The prevalence of HIV and HBV ranks the first position in the country [23]–[26]. In 2003, Wang et al. [23] studied the prevalence of HTLV-1/2 among blood donors in Henan and Hubei province, and found no HTLV-1/2-positive cases. Little information on HTLV-1/2 prevalence has been found in other areas of Central China. The aims of this study were to estimate the seroprevalence and to characterize the epidemiologic patterns of HTLV-1/2 infections in Central China; to detect the potential differences among blood donors, patients with malignant hematological diseases, and high risk groups; to further identify the possible high risk factors for the transmission of HTLV-1/2 in Central China.

## Methods

### Ethics Statement

The study design was approved by the Committee on Human Research of Zhengzhou University located in Zhengzhou, China. All adult participants and the parents/legal guardians on behalf of their children (5–18 years) signed a written informed consent sheet, and were informed about the purpose and procedures of the study. All participants were free to withdraw from the study at any time without further obligation.

### Study Population

From Jan 2008 to Dec 2011, a cross-sectional study was performed to assess the seroprevalence of HTLV-1/2 infection among different population in Henan and Hubei, China. A total number of 5480 blood samples were collected and divided into three groups. The random samples of 3548 voluntary blood donors were recruited from 2 blood banks (Henan Red Cross Blood Center and Wuhan blood center of Guangzhou military). All these donors fulfilled the criteria for blood donation. The 908 cases were patients with malignant hematological diseases (MHDs) attending the First Affiliated Hospital of Zhengzhou University and Wuhan General Hospital of Guangzhou Military, which were diagnosed by the Hematology Unit, including acute lymphocytic leukemia (ALL, 149/908), acute myeloid leukemia (AML, 361/908), chronic lymphocytic leukemia (CLL, 12/908), chronic myelogenous leukemia (CML, 77/908), non-Hodgkins lymphoma (NHL, 287/908), and Hodgkins lymphoma (HL, 22/908). The remaining 1024 cases were high risk group (HRs), including injecting drug users (IDUs, 81/1024), female sex workers (FSWs, 279/1024), men who have sex with men (MSM, 69/1024), former paid blood donors (PBDs, 77/1024) and patients attending clinics for sexually transmitted infections (STIs, 518/1024) in Zhengzhou and Wuhan, China. All samples went through a standard screening for antibodies to HIV (anti-HIV), HBV (HBsAg), HCV (anti-HCV), and *T. pallidum* (Syphilis ELISA IgG + IgM).

### Screening Assays

Participants were screened for the presence of HTLV-specific antibodies using an enzyme-linked immunosorbent assay (ELISA: Beijing Wantai Biological Pharmacy Enterprise Co., Beijing, China). Briefly, 212 amino acids from the envelope region (aa 185–396) of HTLV type 1 fused to 26 amino acids from the envelope region (aa 185–210) of HTLV type 2 were expressed in *E. coli*. The purified recombinant polypeptide was labeled with horse radish peroxidase (HRP) and coated onto microtitre plate. The assays were performed according to the manufacturers' instruction, which indicates that serum samples that yielded an OD/cut-off ≥1.0 are considered positive for HTLV-1/2 antibodies. Reactive samples were retested in duplicate with the same kit.

All repeatedly reactive samples were further confirmed by Western blot assay (HTLV blot 2.4, Genelabs Diagnostics, Science Park, Singapore) in accordance with the manufacturer's criteria. Seroreactivity was interpreted according to the stringent criteria indicated by the manufacturer. A Western blot test was scored as HTLV-1 positive only if bands for the Gag (p19 with or without p24) and two Env (GD21 and rpg46-1) bands were detected; it was scored as HTLV-2 positive if bands for the Gag (p24 with or without p19) and two Env (GD21 and rpg46-2) were found. The presence of p24, p19, and GD21 was considered as HTLV positive but nontyped. Samples that demonstrated reactivity to Gag (p19 and p24) and Env (GD21, rgp46-1 and rgp46-2) were defined as HTLV-1- and HTLV-2-positive. Any other pattern of bands was considered indeterminate.

### Extraction of DNA and Polymerase Chain Reaction

For sequencing of full proviral genomes, proviral DNA was prepared form peripheral blood lymphocytes of the HTLV-1 positive individual with non-Hodgkin's lymphoma. Each major coding regions and the long terminal repeat (LTR) were amplified with Primes STAR HS DNA Polymerase (Takara Biotechnology Co., Dalian, China) [27]. All overlapping PCR fragments covered the complete HTLV-1 genome. The PCR products were purified and directly sequence (Sangon Biotech, Shanghai, China). At the same time, purified products were cloned into the pGEM-T vector (T/A Cloning; Promega), and sequence data were generated from clones (Sangon Biotech, Shanghai, China). DNA sequences were assembled and edited using Sequencher software version 3.1 (Genecodes, inc., Ann Arbor, MI, USA).

### Statistical Analysis

Chi-square test and Fisher's exact test were used to compare proportions. Logistic regression was applied to estimate the magnitude of associations to risk factors for HTLV infection, expressed as odds ratios with 95% confidence intervals. All statistical analyses were performed by using SPSS software version 17.0 (SPSS Incorporated, Chicago, IL, USA).

### Phylogenetic Analysis

The complete genome sequence of HTLV-1 strain newly reported here has been deposited in GenBank database under accession number KF807984.

All 18 publicly available full-length HTLV-1 proviral genome sequences were downloaded from GenBank and aligned with the new proviral sequence. The geographic locations of recovery and accession numbers of the GenBank sequences are as follows: Germany: AF042071; Caribbean: M86840, D13784; USA: AF139170, NC_001436, AF033817; France: L36905; Brazil: AY563953, AY563954; Canada: HQ606137, HQ606138; Japan: L03561, U19949, J02029, AB513134; China: AF259264; Central Africa: JX507077; Solomon Islands: L02534. All of these sequences have been shown previously to belong to the so-called “Cosmopolitan” HTLV-1 subtype A [27], except the Solomon Islands (L02534) and Central Africa (JX507077), which belongs to HTLV-1 subtype C and B, respectively [28], [29]. The full-genome alignment was 8303 nucleotides (nt) in length.

We also performed the phylogenetic analysis on gp46 sequence (683 nt). These isolates were representative of subtype A: D13784 and AY604934 (French Guiana) for the West African subtype; AF 438763 (Japan), M37301 (Japan) and J02029 (Japan) for Japanese subtype; AF165067 and AF165068 (Fujian province, China), AY604935 (Iran), U32556 (British Columbia), M69044 (African), AY604894 (Franch Guiana) and L76061 (Caribbean) for the Transcontinental subtype. The representative isolates of genotype B are L26586, AY604933 (Gabon), AF091494 (Gabon); and the representative isolates of genotype C are L02434 (Melanesia), L02533 (Melanesia), and M92818 (Australian aboriginal).

The sequence alignment were made using Clustal W program; gaps were removed, and distance-based trees were generated by using the Kimura two-parameter model in conjunction with the neighbor-joining method in the MEGA program (version 5.2). The reliability of the final topology of the trees was tested with 1000 bootstrap replicates.

## Results

### Characteristics of the Study Population

A total of 5480 subjects were enrolled in this study: 3296 (60.1%) were males and 2184 (39.9%) were females. The average age ±SD of the study population was 29.21 years ±12.03 (range  = 3–72 years). Infection with HIV, HBV, HCV, and *T. pallidum* was detected in 12 (0.22%), 272 (4.96%), 357 (6.51%), and 149 (2.72%) of study participants, respectively. Demographic characteristics and co-infection with HIV, HBV, HCV and *T. pallidum* are shown in Table 1.

**Table 1:** Demographic Characteristics Among Samples.

Group	No. tested	Male	Female	<20y	20–40y	>40y	HIV	HBV	HCV	TP
Donors	3548	2250	1298	266	2762	520	1	24	15	11
MHDs	908	517	391	185	219	504	1	133	221	0
HRs	1024	529	495	183	552	289	10	115	121	138
All	5480	3296	2184	634	3533	1313	12	272	357	149

Note: MHDs, patients with malignant hematological diseases; HRs, high-risk groups; HIV, human immunodeficiency virus; HBV, hepatitis B virus; HCV, hepatitis C virus; TP, *T. pallidum*; y, years.

### Serologic Results

**Figure 1:**
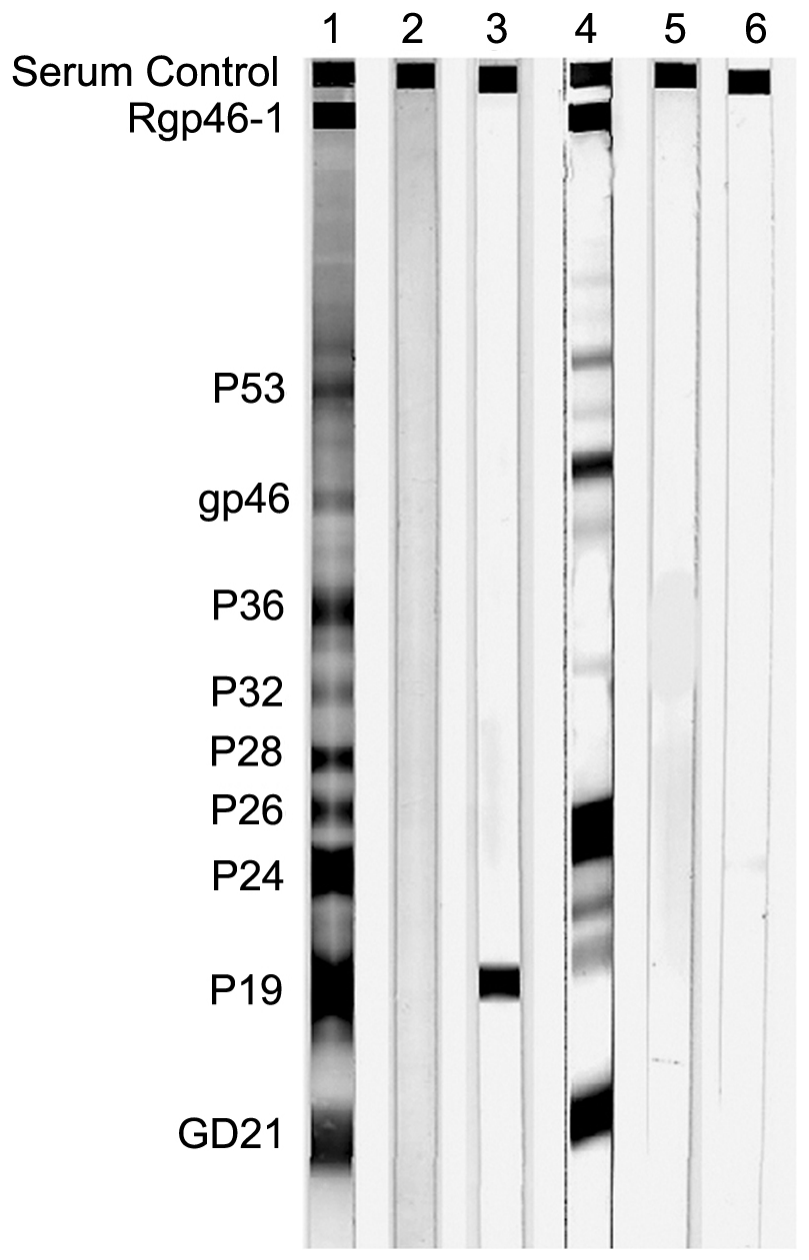
HTLV WB serologic pattern of samples. Lanes 1 and 2: control sera (positive and negative). Lanes 3: indeterminate WB result; lane 4: positive sample result; lane 5 and 6: negative results. Reactivity to HTLV-specific proteins is indicated on the left.

In total, 17 of 5480 samples in this study were positive for anti-HTLV antibody using the ELISA assays. Seven of 17 samples were confirmed positive for anti-HTLV-1 antibody and three of 17 samples positive for anti-HTLV-2 antibody using the immunoblot (data not shown). Figure 1 shown the four results obtained on WB 2.4 analyses among 17 ELISA positive samples. Among them, one HTLV-1 positive sample was defined as presence of GD21, p19, p24, gp46 and rgp46-1 (lane 4). One serum presented the band of p19 and be defined as indeterminate (lane 3). The remaining two samples have no band to present. (The result of WB 2.4 analyses for HTLV-2 positive samples was not shown).

Table 2 shows the final infection of HTLV-1/2 for each study groups. A total of 10 positive cases were detected by WB with prevalence for HTLV-1/2 infection. Among them, 7 were HTLV-1 (0.13%), and 3 were HTLV-2 (0.05%) cases. There was no significant difference in seroprevalence between HTLV-1 and HTLV-2 (*p* = 0.21). Of the 7 HTLV-1 positive cases, 2 were blood donors (0.06%), 1 was lymphoma/leukemia patient (0.11%), and 4 were high risk population (0.39%). The prevalence of HTLV-1 in high risk group was significantly higher than in blood donors (*p* = 0.03). HTLV-2 prevalence was 0.03% (1/3548) among blood donors and 0.2% (2/1024) among high risk group. None of 908 lymphoma/leukemia patients had HTLV-2 antibodies. No significant difference was found in HTLV-2 seroprevalence between blood donors and high risk group (*p* = 0.10).

**Table 2:** Prevalence of HTLV-1/2 Among Different Population, Gender and Age Groups.

Characteristics	No. tested (%)	HTLV-1 positive (%)	*p*	HTLV-2 positive (%)	*p*
Population			**0.03**		0.10
Donors	3548 (64.7)	2 (0.06)		1 (0.03)	
MHDs	908 (16.6)	1 (0.11)		0	
HRs	1024 (18.7)	4 (0.39)		2 (0.20)	
Gender			0.82		0.72
Male	3296 (60.1)	5 (0.15)		2 (0.06)	
Female	2184 (39.9)	2 (0.09)		1 (0.05)	
Age (years)			0.45		0.68
<20	634 (11.6)	1 (0.16)		0	
20–40	3533 (64.5)	3 (0.08)		2 (0.06)	
>40	1313 (24.0)	3 (0.23)		1 (0.08)	
All	5480	7 (0.13)		3 (0.05)	0.21

Note: MHDs, patients with malignant hematological diseases; HRs, high-risk groups.

Gender groups and age groups show no significant association with the infection of HTLV-1/2. The prevalence of HTLV-1 and HTLV-2 among male population (0.15% and 0.06%) tended to be higher than that among female population (0.09% and 0.05%) (*p* = 0.82, *p* = 0.72). In addition, majority of HTLV-1 infection were found among >40 years age group,yielding a prevalence of 0.23%, followed by <20 years age group and 20–40 years age group with prevalence of 0.16% and 0.08%, respectively (*p* = 0.45). There was no HTLV-2 positive individuals in <20 years age group, although the prevalence among 20–40 years and >40 years age group were 0.06% and 0.08% (*p* = 0.68).

### Co-infection with HIV, HBV, HCV, and *T. pallidum*

Co-infection of HTLV-1 and 2 with sexually and parenterally transmitted agents is shown in Table 3. Among the 7 HTLV-1positive cases, 6 were co-infected with HBV (2.2%), 2 with HCV (0.56%), and 1 with HIV (0.83%). All the 3 HTLV-2 positive individuals were also infected with HBV (1.1%). One of them also displayed HCV co-infection (0.28%). Co-infection of HTLV-1/2 with *T. pallidum* was not found in study population. The infection rates with HIV, HBV and HCV among HTLV-1 positive individuals were significantly higher than that in HTLV-1 negative individuals (*p*<0.05). On the other hand, it shows statistically significant difference between various pathogens co-infection among HTLV-1 positive cases (*p* = 0.03).

**Table 3:** Co-Infection of HTLV-1/2 Infection with HIV, HBV, HCV and TP.

Agent	No. tested (%)	HTLV-1 positive (%)	*p*	HTLV-2 positive (%)	*p*
HIV			**0.00**		
Positive	12 (0.22)	1 (8.3)		0	
Negative	5469 (99.8)	6 (0.11)		3 (0.05)	
HBV			**0.00**		
Positive	272 (5.0)	6**#** (2.2)		3**#** (1.1)	
Negative	5208 (95.0)	1 (0.02)		0	
HCV			**0.02**		0.47
Positive	357 (6.5)	2**#** (0.56)		1**#** (0.28)	
Negative	5213 (93.5)	5 (0.10)		2 (0.04)	
TP					
Positive	149 (2.7)	0		0	
Negative	5331 (97.3)	7 (0.13)		3 (0.06)	
All	5480	7 (0.13)		3 (0.06)	

Note: HIV, human immunodeficiency virus; HBV, hepatitis B virus; HCV, hepatitis C virus; TP, *T. pallidum*; **#** One participant was co-infected with HBV and HCV.

### Risk Factors Analysis

In the univariant analysis gender, age showed no significant association with the risk of HTLV-1 infection. HTLV-1 prevalence rate appeared to be higher among high risk group, and HTLV tended to be found in individuals positive for HIV, HBV or HCV antibodies, with variable tendency. To further identify independent risk factors, all variables from the univariate analysis were entered into multiple logistic regression models (Table 4). In this analysis, HTLV-1 positive status was associated with HIV (OR = 44.72, 95% CI = 2.98–670.86, *p* = 0.006) and HBV (OR = 107.71, 95% CI = 12.87–908.43, *p* = 0.000). No independent risk factors were found to be associated with HTLV-2.

**Table 4:** Analyses by Risk Factor.

Risk factor	β	S.E	x^2^	*p*	OR	95% CI
HIV	3.80	1.38	7.57	0.006	44.72	2.98–670.86
HBV	4.77	1.09	18.50	0.000	107.71	12.78–908.43

Note: HIV, human immunodeficiency virus; HBV, hepatitis B virus; β, parameter estimation value; S.E, standard error; x^2^, test statistics; OR, odds ratios; 95% CI, 95% confidence intervals.

### Sequence Analysis

**Figure 2:**
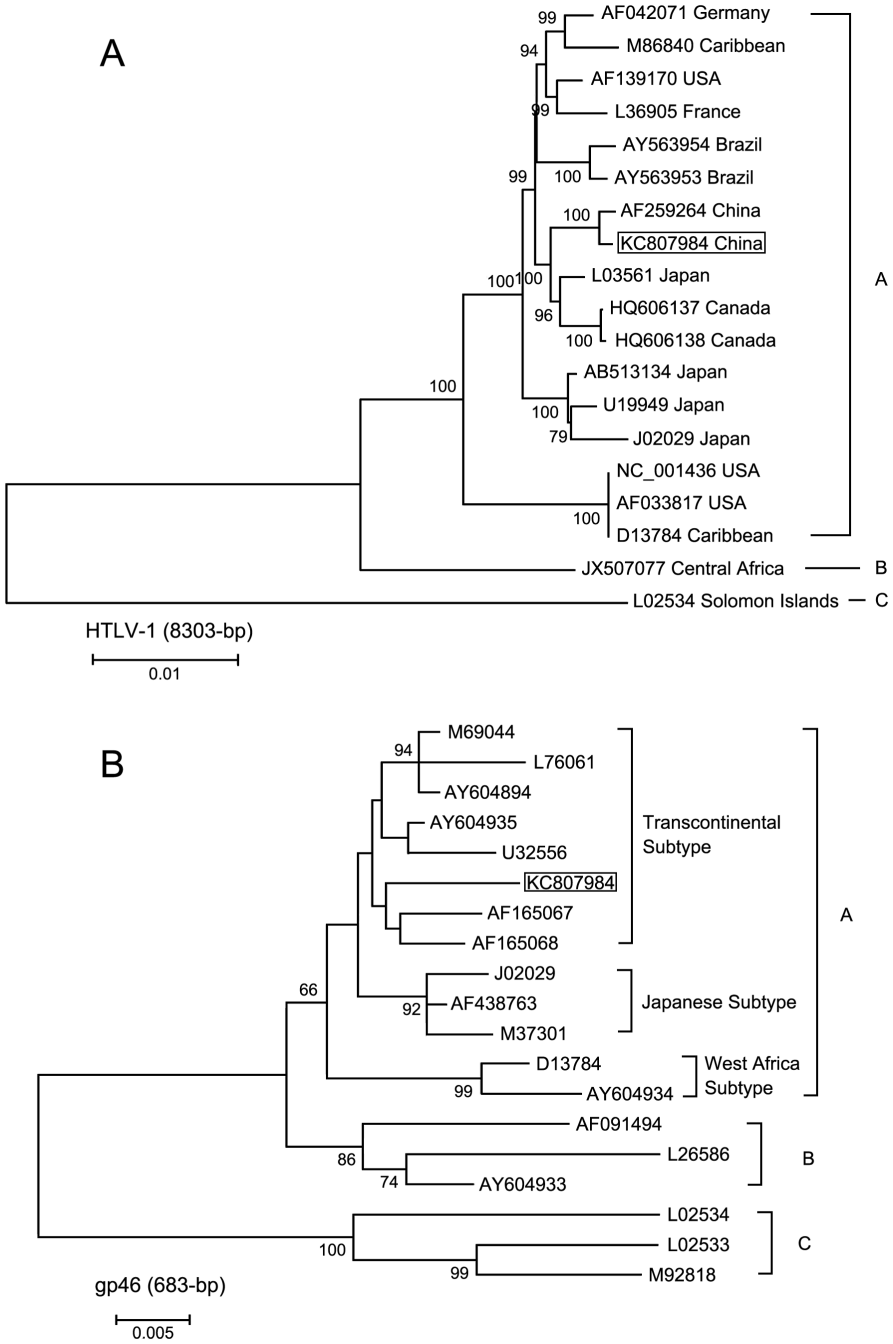
Phylogenrtic tree constructed using complete HTLV-1 genome (8303 bp) (A) and HTLV-1 gp46 (683 bp) (B) sequences by neighbor-joining analysis. Sequence generated in the current study is shown in box. Sequences AF165067 and AF165068 also were derived from samples from China (Fujian province). Support for the branching order was determined by 1000 bootstrap replicates; only values of 60% or more are shown. A =  subgroup A; B =  subgroup B; C =  subgroup C.

The length of the complete genome sequence reported here was 9034 bp. In the phylogenetic tree, this new proviral sequence KC807984 can be recognized in the Genotype A. This new strains formed a tight clade with other genome form China (AF259264) and clustered with genomes from Japan (L03561) and Canada (HQ606137 and HQ606138) (Figure 2A).

In Figure 2B, three subtypes can be recognized in the Genotype A: the West Africa, Japanese, and Transcontinental subtypes. The isolation from our study clearly cluster with members of the Transcontinental subtype and not the Japanese subtype.

## Discussion

This is the first large-scale cross-sectional study to evaluate the prevalence and epidemiological characteristics of HTLV-1/2 infections and co-infections with other pathogens among different population in Central China. Our findings indicate that the final HTLV-1/2 seroprevalence rate was 0.18% (10/5480), yielding a prevalence of 0.13% for HTLV-1 and 0.05% for HTLV-2. The lowest prevalence of HTLV-1/2 infection is found in blood donors (0.06% and 0.03%). However, population of high risk group shows the high level of HTLV-1 and HTLV-2 infection (0.39% and 0.2%, respectively). Moreover,these findings suggest that HTLV-1/2 seroprevalence is associated with HIV, HBV and HCV infections. Among 10 HTLV-1/2 positive cases, 9 were co-infected with HBV, 3 with HCV and 1 with HIV.

Due to inadequate treatment options for TPS/HAM or ATL and lack of an effective vaccination, prevention of new HTLV infections is currently only possible by restricting transmission, either blood transfusion or sexually intercourse. In this regard, knowing the HTLV prevalence status seems to be the most effective way to reduce the transmission of HTLV. China is non-endemic area for these viruses. In 1980s, Zeng et al. [21] conducted an epidemiological investigation in 10,013 blood samples from 28 provinces of China, and found 8 had antibody to HTLV-1. 3 were Japanese living in China and 2 were from Taiwan. 2 were Chinese females from mainland China which husband were Japanese and Taiwan with seropositive. Only one ATL patient was Chinese but often lived in Japan. There was no native citizen infected with HTLV. However, some recent survey show that HTLV infection cases have been found in some provinces of the country, and the prevalence rate in health blood donors were 0.06–1.27% [20], [22], [23]. The coastal areas of Fujian province and Guangdong province show higher prevalence. In this study, we found 2 HTLV-1 positive cases and 1 HTLV-2 positive case among 3548 blood donors, the prevalence was higher than previous investigations in this area and contemporaneous investigations of the other regions [23], [30]. Although 1 case (HTLV-1) came from Wuhan blood center and the other 2 cases came from Henan Red Cross Blood Center, we could not determine the exact origin of these HTLV positive individuals due to the lack of the native place information for each sample.

ATL is one of the major HTLV-1 associated diseases. 2–6% HTLV-1 carriers could develop ATL if infection occurs in early childhood [31]. Barrientos and his colleagues [32] tested HTLV-1/2 antibody among 88 patients with malignant hematological diseases in Southern Chile. The viral prevalence was 18% among these patients. In Okinawa, Japan, an endemic HTLV-1 region, Miyagi et al. [33] found an HTLV-1 prevalence of 26.1% in 88 cases of non-Hodgkin's lymphoma. In 1997, it has been reported that the seroprevalence was 28.9% among patients with T-cell lymphoid malignancies in Brazil [34]. In this study, only one case was found to be HTLV-1 antibody positive among 908 lymphoma/leukemia in Central China, the prevalence rate was 0.11%, which was lower than that reported in other countries. Although this positive case was 48 years old male with non-Hodgkin's lymphoma, there has no diagnosis index of ATL for this patient. It suggests that HTLV-1 infection is not the main cause of malignant hematological diseases in Central China [35]. The HTLV-1 infection of this patient may be related to repeated injection blood product during the treatment of diseases. High molecular weight DNA was obtained from one HTLV-1 infected patient and was subjected to PCR to obtain the complete proviral genome (9034 bp) and the sequence analysis was performed by phylogenetic tree. Molecular studies on complete HTLV-1 genome based on phylogenetic analysis have demonstrated that two HTLV-1 isolates from China, one from this study and another from Fujian province, all belong to genotype A. We observed a close phylogenetic affinity between these two China strains and three strains from other region: one from Japan (L03561) and two from Canada (HQ606137 and HQ606138).

Like other sexual transmitted diseases, some high risk behaviors cause the spread of the virus, including sexual contact without protection with multiple partners, sharing injecting equipment, unsafe blood product transfusion, etc. Therefore, high risk behavior population could be the source of HTLV-1/2 spread in non-endemic regions, and cause co-infection with other sexually transmitted pathogens due to the same modes of transmission [36]–[39]. The study of Giuliani et al. [40] in Italy found the prevalence was 0.6% both for HTLV-1 and HTLV-2 among 1457 high risk population. Further analysis showed that *T. pallidum* were associated with HTLV-1 infection; IDUs play an important role in HTLV-2 transmission; and the significant high rate of HTLV-1/2 infection has been found in HCV/HIV/HBV antibody positive individuals. In Argentina, Berini et al. [41] surveyed the infection of HTLV-1/2, HIV, HBV, HCV and *T. pallidum* in different high risk groups, the prevalence for HTLV-1/2 was 2.4%. The highest prevalence of HTLV-1/2 was found among IDUs (19.1%) and the lowest was MSM (0.4%). High rate (63%) of co-infection between HTLV-1 and HBV was observed. In this study, the participants of high risk group including IDUs, FSW, STIs, MSM, and PBDs. 2 HTLV-1 positive cases were found in FSW and 2 were from STIs. The prevalence rate was the highest in high risk population compared to those two groups of the blood donors and MHDs. The result was in agreement with Wang et al. [42] report. Two HTLV-2 cases were IDUs, suggesting that parenteral transmission maybe important for the spread of HTLV-2. This finding is also consistent with other studies [43], [44]. In addition, of the 7 HTLV-1 cases, 6 were co-infected with HBV, 2 with HCV, and 1 with HIV. In the multivariate analysis, HBV and HIV remained independently associated with HTLV-1.

This study suffered from some limitations. First, due to the nature of cross-sectional study, the associations reported herein between the infections and the relate risk factors may not be related to a cause-effect manner. Second, the limitation of the low prevalence of the HTLV cases in Central China and the inadequate size of the study samples may have caused the lack of statistical significance of some associations, although in the presence of high ORs. Third, the convenience sampling may have led to bias and therefore either underrepresentation or overrepresentation of true prevalence. Last, this study was conducted only in provincial capital cities of Henan and Hubei. Thus, we were unable to evaluate any geographical region characters among any high risk factors across the country. However, we still believe that we could evaluate actual prevalence and associated risk factors in a reliable manner.

In summary, our study reveals the seroprevalence of HTLV-1/2 among different population samples in Central China, which shows a high prevalence rate among high risk group, and low rate among blood donors and MHDs. The infection of HTLV-1/2 has not been found in malignant hematological diseases. HIV, HBV, and HCV were the common risk factors associated with HTLV-1/2 infection, suggested that further study should be carried out in specific population, aiming to prevent the virus spreading. Our results can also provide theoretical basis for revise standard of voluntary blood donation in this area.
