# Correction: Epidemiological Analysis of HTLV-1 and HTLV-2 Infection among Different Population in Central China

**DOI:** 10.1371/annotation/98da58ce-6d7c-4fc5-ba3e-fa8ddcf8479c

**Published:** 2013-07-15

**Authors:** Yunyun Ma, Shangen Zheng, Na Wang, Yu Duan, Xinyu Sun, Jing Jin, Wenqiao Zang, Min Li, Yuanyuan Wang, Guoqiang Zhao

The accession number listed in the Phylogenetic Analysis section of Methods is incorrect. The correct accession number is KC807984. 

